# Dietary Inositol Reduces Fearfulness and Avoidance in Laying Hens

**DOI:** 10.3390/ani9110938

**Published:** 2019-11-08

**Authors:** Eugenia Herwig, Henry L. Classen, Carrie L. Walk, Mike Bedford, Karen Schwean-Lardner

**Affiliations:** 1 Department of Animal and Poultry Science, University of Saskatchewan, Saskatoon, SK S7N 5A8, Canada; 2 AB Vista, Marlborough, SN8 4AN, UKMike.Bedford@abvista.com (M.B.)

**Keywords:** myo-inositol, phytase, behaviour

## Abstract

**Simple Summary:**

Brain inositol is known to affect memory, and the incidence of depression, anxiety, and obsessive-compulsive disorder in mammals. Phytate, a naturally occurring inositol ester in plants, binds other nutrients, making them unavailable for digestion. The addition of phytase, the enzyme capable of hydrolyzing phytate, to diets increases the release of both inositol and nutrients for absorption in the chicken digestive tract. In this study, we assessed how dietary phytase or pure inositol affected laying hen behaviour, fearfulness, aggression, and stress levels. To increase the probability of seeing effects, hens were not beak treated and were fed two balanced protein levels differing in digestible amino acid sufficiency. Inositol did not affect stress levels, as measured by heterophil-to-lymphocyte ratio, or the number of hen comb or skin lesions. However, regardless of the source, pure inositol or phytase derived inositol reduced the number of feathers in the vent area, suggesting an increase in feather pecking. Pure inositol reduced fearfulness in laying hens, but phytase-derived inositol did not.

**Abstract:**

Myo-inositol (inositol) affects memory, and the incidence of depression and anxiety in mammals. An experiment was designed to determine if pure inositol (0.16%), or high levels of phytase (3000 FTU/kg) affect the behaviour of fully beaked Lohmann LSL lite hens fed amino acid sufficient (19% crude protein (CP)) and deficient diets (16% CP), from 19 to 59 weeks of age. The data collected included live-scan behaviour observations and novel object (NO) tests (both at 1, 10 and 40 weeks of the trial); heterophil-to-lymphocyte (H/L) ratios (week 1 and week 40 of the trial); end of trial feather cover, and comb and skin lesions; and daily mortality. Reducing CP increased sitting by 2.5%. Inositol, but not phytase, reduced the latency to peck at the NO by 300 sec. Inositol reduced vent feather cover by 12% and tended to increase mortality by 13%. No effects on H/L ratio, and comb or skin lesions were found. In conclusion, regardless of the source, inositol reduced vent feather cover, while it tended to increase mortality. Only pure inositol reduced fearfulness in laying hens.

## 1. Introduction

Chickens have a limited ability to break down dietary phytate (myo-inositol hexakisdihydrogen phosphate (IP6); [1]). Thus, exogenous phytase is added to poultry diets to increase phosphorus release and diminish IP6-related anti-nutritional effects. In recent years, the industry trend had geared towards supplementing poultry diets with 1500 FTU/kg or more. High levels of phytase have been shown to break down approximately 90–95% of phytate in broiler diets [2,3], and as a consequence, improving diet digestibility and bird performance [4,5]. Although the targeted objective of phytase is the release of phosphorus and improvement in nutrient utilization, myo-inositol (inositol), a hydrolysis by-product, is also produced. High levels of phytase are particularly efficient in completely de-phosphorylating IP6, as there is a positive linear relationship between the level of phytase supplementation and inositol concentration in chicken digesta [6]. The presence of inositol in chicken digesta might have physiological effects, as shown by Walk et al. [5], who reported a positive correlation between inositol concentration in gizzard digesta and feed conversion ratio and body weight gain in broilers [7].

Inositol is a cyclical isomer of glucose, which is involved in many physiological processes, such as insulin sensitivity (inositol pyrophosphate), lipid and cell metabolism (various inositol forms), transduction of brain signals (inositol triphosphate), and modulation of circadian rhythms (IP6; [7,8,9,10]). Most of the inositol body demands can be met by endogenous synthesis, and inositol transporters (sodium/myo-inositol I and II, and H+/myo-inositol transporters) can be found in a variety of tissues, such as kidney, brain, pancreas, heart, skeletal muscle, lung, and the digestive tract [11]. In addition to endogenous synthesis, inositol can be derived from dietary sources, such as the action of exogenous phytase on dietary phytate. The evidence of inositol release by the latter is shown by a finding that feeding high levels of phytase to broiler chickens upregulates the expression of the small intestine H+/inositol co-transporter [6].

Inositol deficiency in the brain increases memory loss and reduces learning capabilities in mice [12], and increases the incidence of depression, anxiety and obsessive-compulsive disorder in rats [13,14]. Due to its ability to modulate a mental state and the amplitude or frequency of the circadian rhythm, inositol has been successfully used to treat psychiatry disorders in humans [10,15,16]. In chickens, a positive correlation has been found between dietary inositol, and serotonin and dopamine plasma concentrations in 21-day old broilers [17]. Serotonin and dopamine are neurotransmitters that are associated with aggression and the reward system, respectively [18]. High levels of serotonin decrease feather pecking [19,20] and aggression [21], but increase fearfulness in chickens [21]. High levels of dopamine are also associated with low feather pecking [19] in chickens, but increase the incidence of aggression [22]. The correlation between these neurotransmitters and inositol reported by Huber et al. [17] suggests that dietary inositol has the capacity of modulating chicken behaviour.

It was hypothesized that high levels of phytase (3000 FTU/kg) or the amount of pure inositol equivalent to a complete de-phosphorylation of dietary IP6 could decrease feather pecking, increase fearfulness, and change the incidence of aggressive behaviour in laying hens. While nutritive and maintenance behaviors are simple to measure, as they comprise a large proportion of the daily behaviour budget of chickens (Bubier, N.E. e.g., [23]), measuring feather pecking, fearfulness, and aggression can be more difficult. Diets can be manipulated to facilitate the measurement of treatment effects in the mentioned variables by decreasing dietary crude protein to increase the incidence of negative behaviors such as feather pecking [24,25]. Thus, the objective of this trial was to determine if dietary inositol or inositol derived from high levels of phytase (3000 FTU/kg) action affected the behaviour of hens fed amino acid-deficient and -sufficient diets.

## 2. Materials and Methods

This work was approved by the University of Saskatchewan’s Animal Research Ethics Board and adhered to the Canadian Council on Animal Care guidelines for humane animal use [26,27].

### 2.1. Experimental Treatments

Diet composition and analyses are presented in Table 1. Two diets were formulated to either meet the requirements (840 mg/h/d D-Lys; [25]; high balanced protein—HBP) or to have a reduced level (80% of HBP diet) of balanced protein (672 mg/h/d D-Lys; reduced balanced protein—RBP). To study the effect of phytase and inositol on laying hen behaviour, the HBP diet was divided into two fractions and inositol was added to one of them, creating two treatments: HBP and HBP-Inositol (HBP-I). Similarly, the RBP diet was divided into three fractions. One fraction was supplemented with inositol (RBP-Inositol or RBP-I) and a second fraction was supplemented with phytase (RBP-Phytase or RBP-P). The RBP-P diet received 3000 FTU/kg of phytase (Quantum Blue 5G, AB Vista, Marlborough, Wiltshire, England), while HBP-I and RBP-I received an amount of pure inositol (0.16%; Inositol powder, BulkFoods, Toledo, OH, USA) equivalent to 60% of diet IP6 hydrolysis and inositol release (40% assumed to be naturally de-phosphorylated). Analyzed composition values of the diets approximate those of the calculated ones.

Diets were provided in two phases (19 to 27 and 28 to 59 weeks of age) based on the estimated feed intake (average feed intake of 95 g/h/d from 19–27 weeks of age and 110 g/h/d thereafter). All diets were formulated to meet or exceed the nutrient requirements for laying hens [28], with the exception of crude protein (amino acids) in the RBP diets. The feed was manufactured at the Canadian Feed Research Centre (CFRC, North Battleford, SK, Canada), and fed in a crumble form.

During the current experiment, data were also collected to assess performance, digestibility, and inositol absorption and bioaccumulation. Bioaccumulation of inositol in egg yolk was equal for the HBP-I, RBP-I, and RBP-P treatments, and these values were higher than in eggs from HBP and RBP, confirming that our treatment formulation successfully met the project objectives [29]. 

### 2.2. Birds and Bird Housing

A total of 360 Lohmann LSL lite fully beaked chicks (Clark’s Poultry, Inc., Brandon, MB, Canada) were floor raised from 0–18 weeks of age in one room at the Poultry Centre at the University of Saskatchewan (Saskatoon, SK, Canada) under environmentally controlled conditions. Birds were fed commercial starter (0–6 weeks of age), grower (6–10 weeks of age), developer (10–16 weeks of age), pre-lay (16–18 weeks of age), and layer feed (18–19 weeks of age; Federated Coop Limited, Saskatoon, SK, Canada) for ad libitum consumption prior to experiment initiation.

At 18 weeks of age, hens were randomly allocated in groups of six to 60 Specht conventional cages (503 cm^2^/bird, 60 cm feeder trough, one lubing nipple drinker). The cages were located in the same tier, and two adjacent cages were considered an experimental unit, resulting in six replications of 12 hens per treatment, arranged in a completely randomized design. The barn was maintained at approximately 21 ℃ and the hens were provided with 12 h of light per d (10 lux) at 19 weeks of age via incandescent bulbs. The day length was increased weekly by one hour until reaching 16 h of light per day at 23 week of age. From then on, the light program (16L: 8D; 10 lux) was maintained until the end of the trial. Feed and water were supplied for ad libitum consumption. The trial started when the hens were 19 weeks of age and lasted for 40 weeks.

### 2.3. Data Collection

During the first, tenth, and last week of the trial, live-scan observations, four times per day for two days, were performed by two observers (one, four, seven and ten hours after the start of the photophase). Each experimental unit was observed once by each observer at each observation time, resulting in a total of 16 observations per experimental unit per hen age. Groups of four cages were simultaneously observed for up to 1 min to determine the activity of each bird in them. The average percentage of time the hens performed each behaviour (Table 2) during the photophase was calculated. One week prior to initiation of observations, inter-observer reliability was assessed between the two observers, and a 97% agreement was obtained. Two days after scan observations, a novel object test was performed to evaluate the effect of treatment on hen fearfulness. A novel object was made using shiny metallic paper of four colors and hung from the cage door. The time until the first hen pecked at the object was recorded. The maximum time allowance for the test was 30 min.

Mortality was collected, weighed, and recorded daily. Cause of death was determined by pathologists at the Prairie Diagnostic Services (Saskatoon, SK, Canada). At 2 and 38 weeks of the trial, blood was collected from three hens per experimental unit to determine heterophil-to-lymphocyte (H/L) ratios as a measure of chronic stress. Birds were caught and blood was collected immediately from the brachial vein into vacutainer tubes containing EDTA. Blood smears were made within an hour of blood collection. Slides were stained using PROTOCOL^TM^ Hema 3^TM^ (Fisher Scientific, Ottawa, ON, Canada) and H/L ratios were determined by counting the number of heterophils and lymphocytes under a 100× oil magnification (microscope B-290TB; Optika; Bergamo, Italy) until a total of 100 cells was reached.

At the end of the experiment, feather coverage, comb damage, and skin lesions were assessed by two independent trained scorers in all live hens. Feather coverage was scored on five body parts: neck, wings, back, vent, and breast. The scoring system ranged from 1 (less than 25% of feather covering) to 4 (more than 75% of feather covering) for each body part, following the procedure reported by Davami et al. [34]. A total score per bird was calculated by adding the average score of the five body parts assessed. Feather cover score data were averaged between scorers before data analyses. Similarly, comb scores were individually scored by counting the number of peck injuries, following an adaptation of the Ali and Cheng [35] scoring system. No pecking damage resulted in a score of zero. A score of one was assigned to single marks, while two or three peck-marks resulted in a score of two. More than three marks resulted in a score of three. A score of four was assigned to severe injuries and extensive comb damage. Skin lesions were assessed based on the Welfare Quality assessment [36]. Each hen was scored individually, and feathers were moved to inspect covered areas. No lesions or less than three pecks was considered a score of zero. Lesions longer than 2 cm or three or more peck-marks was assigned a score of one. A score of two was recorded for injuries longer than 2 cm.

### 2.4. Chemical Analysis

A sample of each of the diets was sent to SGS Agrifood Laboratories (Guelph, ON, Canada) to be analyzed. Dietary analysis included moisture, protein (N × 6.25), calcium and phosphorus following methods A202, A203a and A204a [37]. Dietary phytate was determined using a high-performance ion chromatography with post column derivatization and UV detection at 290 nm [38].

### 2.5. Statistical Analyses

The experiment was arranged as a complete randomized design. Behaviour scan sampling data and novel object test results were analyzed with a repeated measures ANOVA (Proc Mixed) of SAS statistical package (version 9.4, SAS Institute, 2004), using dietary treatments as the main factor. Both factors, time and dietary treatments, were considered fixed effects. The remaining data (H/L, mortality, feather cover and comb and skin lesions) were analyzed with a one-way ANOVA (Proc Mixed). For all data, the following orthogonal contrasts were conducted: an assessment of protein level (HBP+HBP-I vs. RBP+RBP-I), a comparison between the effect of inositol under high protein levels (HBP vs. HBP-I), the overall impact of inositol (HBP-I+RBP-I vs. HBP+RBP), and an assessment of the effect of phytase and inositol under reduced protein level (RBP-P vs. RBP-I). All the data except for feather cover and lesions were log+1 transformed before analyses to reach normality. All differences were considered significant at *p* ≤ 0.05 and trends were considered when 0.10 ≥ *p* > 0.05.

## 3. Results and Discussion

**Figure 1:**
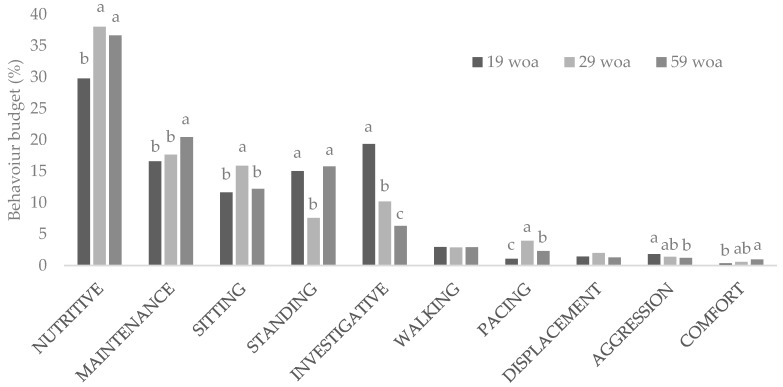
Percentage of time laying hens housed in conventional cages performed each behaviour category during the photophase at 19, 29 and 59 weeks of age. Behavioral categories analyzed included: nutritive (feeding and drinking behaviors), maintenance (preening, sham-dustbathing, ruffling), sitting (sitting and sleeping), standing, investigative (gentle feather pecking and object pecking), walking, displacement, aggression (aggressive pecking, severe pecking and fighting), and comfort (wing or leg stretching, flapping and tail wagging). Woa: week of age. a,b,c: letters indicate time differences in the incidence of the behaviour.

Throughout the trial, the majority of the daytime behaviour budget was spent performing nutritive behaviors (34.8%). Other highly displayed behaviors were those related to maintenance (18.2%), sitting (13.3%), standing (12.8%) and investigation (11.9%), while walking, pacing, displacement, aggression and comfort behaviors comprised the remaining 9.0% of the behaviour time budget (Figure 1). Week of age affected the incidence of every behaviour with the exception of displacement (Table 3). While the time spent performing some behaviors appeared to increase with age (e.g., nutritive, maintenance and comfort), others decreased (investigative and aggression). Figure 1 shows a reduction in the behaviour budget assigned to investigative or aggressive behaviors as hens become older. These reductions in aggression and investigative behaviour are not surprising in a stable cage environment, in which the lack of resources reduces the motivation to investigate and the peck order was probably established during the first weeks of the trial [39,40]. Similar results on the incidence of object pecking with age have been reported by Anderson and colleagues [41]. The authors associated this drop in object pecking with the lack of environmental novelty.

Laying hen daily feed intake generally increases until birds are approximately 25 weeks of age, after which it remains constant [42]. This pattern may explain why the time spent performing nutritive behaviors increased after the first observation (19 weeks of age). The apparent increase in comfort behaviour with age is a positive sign that hens in this trial had enough space to express this type of behaviour [43]. However, given the low incidence of comfort behaviour in the current experiment, and the fact that only a trend was found, its biological significance should be questioned. Although preening, sham-dustbathing and ruffling all changed in the same manner, preening accounted for the largest proportion of the maintenance behaviors. The increase in maintenance behaviors is consistent with previous reports which indicate that both the number of bouts and the time spent preening increases with age [44]. Egg-laying is frequently associated with pacing, particularly when hens are housed in environments that do not support this behaviour [45]. Laying hens reach the peak of lay at around 30 weeks of age [42], which matches the increase in pacing observed during the 29 week of age observation.

**Figure 2:**
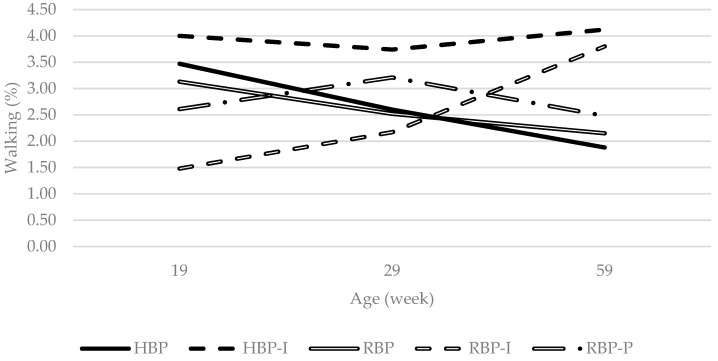
Interaction between dietary treatment and age on the incidence of walking in the Lohmann LSL lite hens behaviour budget. HBP: High balanced protein; RBP: Reduced balanced protein; I: Inositol; P: Phytase.

An interesting point is a significant interaction found regarding the percentage of time birds spent performing walking behaviour (Figure 2). Albentosa et al. [44] reported that walking decreased with age in cage-housed hens and both HBP and RBP treatments (without inositol supplementation) followed the same pattern. In contrast, hens provided with additional inositol either did not change walking activity (RBP-P, HBP-I) or demonstrated an increase (RBP-I) in walking with age. The failure of inositol fed hens to follow a decreasing pattern of walking may relate to its effects on animal activity. Mammalian studies have observed an increase in activity levels (walking or swimming) when animals were fed inositol [13,46,47]. However, others have found no such effect [48,49]. The discrepancy in the literature may relate to inositol dosage and/or animal physiological state. Supporting an effect of inositol on activity, orthogonal contrast analysis in the current study found that inositol tended to increase walking (*p*_inositol_ = 0.066).

A decrease in activity levels (lassitude) in mammals have been associated with symptoms of depression and apathy [50]. Psychiatric research conducted in humans has suggested an inverse association between brain inositol levels and apathy [16]. Similarly, Sprague-Dawley male rats with chronic intraperitoneal (1.2 g inositol/kg) or oral inositol supplementation (25–30 g/day) showed increased activity levels when using the forced swim or the reserpine-induced immobility, a pharmacologically induced model, tests when compared to control rats [47]. The results of the current study indicate that continuous dietary inositol supplementation more than a hundred times lower than that used in Einat and collaborators study (0.18 g/day based on a measured average daily feed intake of 118 g/day per hen) might increase or maintain high levels of walking in laying hens.

Hens fed RBP diets spent less time pacing and more time sitting than hens fed HBP diets (Table 3). The HBP diets in the current trial met or surpassed all the Lohmann nutritional requirements for LSL-lite hens, while the RBP diets had a 20% reduction in amino acid balanced CP. It is possible that the relative deficiency of protein induced a change in behaviour, as similar behavioral observations have been reported for energy deficiency. During fasting it is common to observe a temporary increase in activity and aggression, which is associated with high plasma corticosterone [51,52] due to hunger. This phase is followed by a decline in activity levels and an increase in resting [53] to reduce energy expenditure. This second phase resembles the pacing-sitting pattern observed in RBP treatments. However, during the current trial, no interaction between time and dietary treatment was found for these behaviors. Since behaviour measurements were done from the first week of the trial, this means that the first phase observed during fasting was not likely present in these birds. Furthermore, hen performance, feed intake (HBP = 117; HBP-I = 120; RBP = 117; RBP-I = 117; RBP-P = 120 g of feed/day/hen; *p*_protein_ = 0.959) and feeding behaviour were not affected by diet, suggesting that the level of protein deficiency was minor and not likely to change behavior in an attempt to conserve protein reserves.

When hens were fed RBP diets, inositol reduced the degree of pacing, while inositol addition to the HBP diets produced the highest observed incidence of pacing in this study (Table 3). The reason why protein level would interact with inositol level is not obvious and it is possible that a high level of variability in a low incidence behaviour, such as pacing, might have resulted in a type I error.

**Figure 3:**
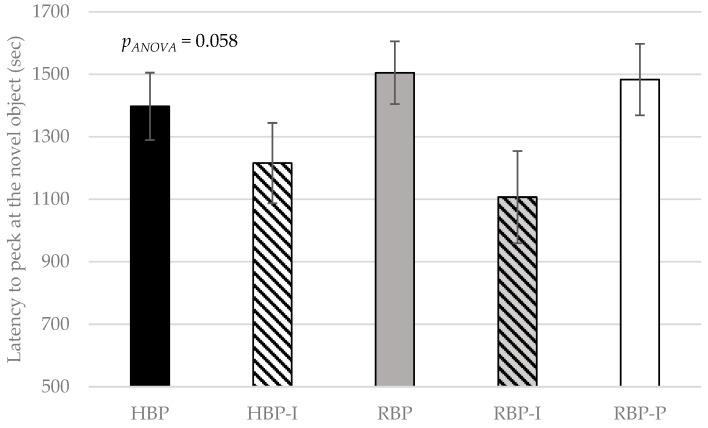
Effect of protein level, inositol or phytase on the latency to peck at the novel object (sec) by Lohmann LSL lite hens. HBP: High balanced protein; RBP: Reduced balanced protein; I: Inositol; P: Phytase.

Latency to peck at the novel object was reduced by dietary inositol supplementation (Figure 3). A trend (*p*_ANOVA_ = 0.058) was found for dietary treatment to affect the latency to peck at the novel object in the repeated measures analysis. An orthogonal contrasts analysis indicated that hens fed diets that included dietary inositol, but not those fed diets with phytase supplementation (*p*_RBP-P vs. RBP-I_ = 0.021), peck at the novel object faster than those on other dietary treatments (*p*_inositol_ = 0.012). This result indicates that pure-dietary inositol might reduce fear responses in hens. In support of this effect is research showing that chronic intraperitoneal inositol injections (1.25 g/kg) increase the time rats spent in the open arms of an elevated plus-maze [13]. Rats are aversive to open spaces, so inositol might have anxiolytic-like effects, allowing them to spend more time in the open arms of a maze. Park et al. [54] found that impairment of inositol polyphosphate multikinase, an enzyme involved in the synthesis of higher polyphosphates, displayed enhanced fear extinction. Impairment of this enzyme would result in higher inositol or lower inositol polyphosphates levels. Thus, these physiological conditions could be associated with a decrease in fear.

No effect of diet on H/L ratios was found. The average H/L ratio found two weeks after the start of the trial was 0.35 (*p*_ANOVA_ = 0.620), while the ratio was 0.43 by trial end (*p*_ANOVA_ = 0.590). This result suggests that none of the dietary treatments affected hen chronic stress levels. What is more, these values approximate those reported in the literature as indicative of low to optimal stress [55,56]. They are also consistent with the low incidence of aggression and pacing, and the increasing performance of comfort behaviors seen with age in the current research. Likewise, these results confirm that the 20% reduction in crude protein level in the RBP treatments was not stressful for the hens as was discussed earlier.

Feather cover, as well as skin and comb lesion results at the end of the experiment, are presented in Table 4. Dietary inositol significantly reduced feather cover in the vent area (*p*_inositol_ = 0.038) at 59 weeks of age. This result is consistent with mortality data, since cannibalism was the most prevalent cause of mortality found in the current experiment, with eight out of 11 cases occurring in the pure inositol supplemented treatments. Immediately after lay, a minor or partial prolapse of the uterus might occur in laying hens, exposing the mucous membrane. It is believed that cannibalism starts as exploratory pecking of this membrane, which quickly escalates causing blood loss and death of the recipient [31]. A decrease in feather cover in the vent area increases the likelihood of cannibalism occurring in laying hens [57,58]. No effect of diet on skin and comb lesions was found in the current experiment, suggesting no differences in aggression levels among treatments.

No beak treatment was applied to the hens employed in the current experiment, which might have increased mortality levels due to cannibalism [59]. Total mortality reached 11.39% (41 hens) in this trial, and it was variable between experimental units. Most hens died from cannibalism (26.83% or 11 hens), with seven cannibalism events occurring in the RBP-I treatment. Other causes of mortality were hemorrhagic fatty liver (21.95%), infectious diseases (12.20%), and osteoporosis (9.76%) among other causes. Dietary inositol tended to increase overall mortality (*p*_inositol_ = 0.069) in the current trial, with no other significant effects of diet on overall mortality found. It is possible that the increase in mortality, particularly due to cannibalism in the inositol treatments (inositol treatments = 8 hens; other treatments = 3 hens), might be associated with the decrease in fear found in the novel object test, as a lack of fear response would reduce avoidance behaviour [60]. Sajja and collaborators [61] found a negative association between high inositol levels in the forebrain and working memory in rats, which could impair the development of appropriate survival mechanisms. The reduction of feather cover in the vent area of hens fed inositol supports this hypothesis. Still, since the highest cannibalism incidence was found in the RBP-I, it is possible that an interaction between pure inositol and a reduced level of amino acids in the RBP-I treatment could have caused such an increase in the incidence of cannibalism in this particular treatment, resulting in a trend for inositol to increase the overall mortality. Further research is necessary to understand this result.

Increased inositol levels in the anterior and posterior cingulate cortex in humans has been associated with anger, aggression and suicidal behaviour [62]. In an assessment done in veterans, a positive correlation was found between suicidal behaviour, as well as aggression, and brain inositol levels. However, in the current experiment, skin and comb lesion scores, like behaviour results, indicate that dietary treatments did not affect the level of aggression among hens (Table 4). The findings reported here suggest that inositol does not directly affect the levels of aggression in laying hens.

## 4. Conclusions

The current experiment was designed to test if dietary inositol or high levels of phytase affected the behaviour of hens fed amino acid-deficient and -sufficient diets. Particular interest was focused on the effect of inositol on feather pecking, fear and aggressive behaviour in laying hens. Our results indicated that, with the exception of vent feather cover, inositol had no impact on the degree of laying hen feather pecking or aggression. Likewise, stress levels, as reflected by H/L ratios and behaviour, were similar among treatments. However, pure inositol, but not IP6 inositol, reduced fear responses, and likely avoidance, in laying hens, which might suggest differences in inositol mode of action depending on its source (pure or IP6 de-phosphorylation) and metabolism.

## Figures and Tables

**Table 1:** Ingredients and calculated composition of high balance protein (HBP) and reduced balance protein (RBP) diets.

Ingredients (%)	19–27 Weeks of Age		28–59 Weeks of Age	
	HBP	RBP	HBP	RBP
Wheat	53.37	63.32	58.82	67.37
Soybean meal	18.81	9.88	14.39	6.72
Canola meal	10.00	10.00	10.00	10.00
Wheat bran	2.00	2.00	2.00	2.00
Canola oil	4.54	3.57	3.88	3.05
Limestone	9.27	9.31	9.22	9.26
Mono-dicalcium phosphate	0.77	0.77	0.52	0.52
Sodium chloride	0.33	0.32	0.30	0.30
Vitamin-mineral premix ^1^	0.50	0.50	0.50	0.50
Choline chloride	0.10	0.10	0.10	0.10
Rovimix Hy-D 62.5 ^2^	0.04	0.04	0.04	0.04
L-Lysine HCl	0.01	0.05	0.03	0.06
DL-Methionine	0.20	0.10	0.15	0.06
L-Threonine	0.05	0.03	0.04	0.02
Xylanase ^3^	0.01	0.01	0.01	0.01
Calculated composition (as is)				
Apparent metabolizable energy (kcal/kg)	2800	2800	2800	2800
Crude Protein	19.92	16.98	18.54	16.00
Digestible lysine	0.84	0.67	0.76	0.61
Digestible methionine	0.48	0.34	0.41	0.29
Digestible methionine and cysteine	0.79	0.63	0.71	0.57
Digestible threonine	0.77	0.52	0.59	0.47
Calcium	3.70	3.70	3.63	3.63
Phytate	1.02	0.97	1.00	0.96
Non-phytate phosphorus	0.35	0.35	0.30	0.30

^1^ Supplied per kilogram of diet: vitamin A (retinyl acetate + retinyl palmitate), 8000 IU; vitamin D_3_, 3000 IU; vitamin E (dl-α-tocopheryl acetate), 25 IU; menadione, 1.5 mg; thiamine, 1.5 mg; riboflavin, 5.0 mg; niacin, 30 mg; pyridoxine, 1.5 mg; vitamin B_12_, 0.012 mg; pantothenic acid, 8.0 mg; folic acid, 0.5 mg; biotin, 0.06 mg; copper, 10 mg; iron, 80 mg; manganese, 80 mg; iodine, 0.8 mg; zinc, 80 mg; selenium, 0.3 mg; calcium carbonate, 500 mg; ethoxyquin, 0.625 mg; wheat middlings, 3822.79 mg. ^2^ 25-Hydroxyvitamin D3: minimum 137.8 mg/kg of premix (DSM, Heerlen, The Netherlands). ^3^ Econase XT 25 (AB Vista, Marlborough, Wiltshire, UK) supplied at 16,000 BXU xylanase/kg of diet.

**Table 2:** Behavioral scan observations ethogram.

Category	Behaviour	Description ^1^
Aggression	Aggressive pecking	Forceful pecking directed to another bird’s head, legs or vent areas.
	Severe feather pecking	Pecking at the plumage of a cage-mate with the intention of removing feathers. Recipient hen can squawk and withdraw.
	Fighting	More than two aggressive pecks between two or more hens.
Comfort	Wing or leg stretching	Extension of wing or leg on one side of the body.
	Other	Flapping, tail wagging, etc.
Displacement	Displacement	Hen stops another hen’s behaviour by taking her location in the cage.
Investigative	Gentle feather pecking	Pecking at the plumage of a cage-mate with little or no damage to it. Feathers are not removed and it is usually ignored by the recipient.
	Object pecking	Pecking at anything other than feed, water, or cage-mate.
Maintenance	Preening	Self-manipulation of feathers on the body using the beak or self-scratching.
	Sham-dustbathing	Side or head-rubbing, vertical wing shaking and side laying with scratching.
	Ruffling	Fluffing up the feathers.
Nutritive	Feeding	Head extended into the feeder, manipulating or ingesting feed.
	Drinking	Head extended to the water line, manipulating the water nipple.
Sitting	Sitting/Sleeping/Laying	A bird sitting with breast on the floor.
Standing	Standing	Hen standing on both legs without doing any other particular action.
Stereotypy	Pacing	Hen restless and walking back and forward without another purpose.
Walking	Walking	Any hen taking more than two steps in a direction and not pacing.

^1^ Behaviour definitions were obtained from van Liere et al. [30], Savory [31], Gabrush [32], and Hunniford and Widowski [33].

**Table 3:** Effect of protein, phytase and inositol on laying hen behaviour budget (%) from 19 to 59 weeks of age.

Behaviour	Diets					SEM	ANOVA *p* Values			Contrasts *p* Values			
	HBP	HBP-I	RBP	RBP-I	RBP-P		Diet (D)	Time (T)	D × T	Protein	HBP vs. HBP-I	Inositol	RBP-I vs. RBP-P
Nutritive	34.45	36.10	32.96	34.42	36.16	0.611	0.201	**<0.001**	0.685	0.147	0.284	0.154	0.260
Maintenance	17.96	17.45	19.09	19.34	17.28	0.441	0.417	**0.001**	0.939	0.115	0.703	0.889	0.128
Sitting	12.08	12.01	14.37	14.65	13.13	0.413	*0.062*	**<0.001**	0.786	**0.003**	0.950	0.899	0.189
Standing	13.93	11.67	12.73	12.58	13.05	0.537	0.452	**<0.001**	0.881	0.864	*0.063*	0.159	0.697
Investigative	12.97	11.96	11.99	11.70	11.06	0.650	0.464	**<0.001**	0.294	0.440	0.138	0.165	0.948
Walking	2.65 ^b^	3.95 ^a^	2.60 ^b^	2.48 ^b^	2.77 ^a,b^	0.155	**0.010**	0.969	*0.050*	**0.019**	**0.005**	*0.066*	0.528
Pacing	2.46 ^a,b^	2.96 ^a^	2.47 ^a,b^	1.67 ^b^	2.56 ^a,b^	0.184	**0.033**	**<0.001**	0.782	**0.021**	0.384	0.389	**0.022**
Displacement	1.37	1.84	1.51	1.39	1.72	0.118	0.592	*0.080*	0.640	0.420	0.148	0.356	0.703
Aggression	1.38	1.54	1.60	1.29	1.54	0.090	0.685	**0.010**	0.205	0.902	0.646	0.514	0.311
Comfort	0.74	0.52	0.67	0.48	0.74	0.069	0.487	**<0.001**	0.753	0.739	0.234	*0.097*	0.202

Means with common letters do not differ significantly among diets. HBP: High balanced protein; RBP: Reduced balanced protein; I: Inositol; P: Phytase; SEM: Standard error of the mean; Protein: HBP + HBP-I = RBP + RBP-I; Inositol: HBP + RBP = HBP-I + RBP-I. Behavioral categories analyzed were: nutritive (feeding and drinking behaviors), maintenance (preening, sham-dustbathing, ruffling), sitting (sitting and sleeping), standing, investigative (gentle feather pecking and object pecking), walking, displacement, aggression (aggressive pecking, severe pecking and fighting), and comfort (wing or leg stretching, flapping and tail wagging).

**Table 4:** Effect of dietary protein, inositol and phytase on laying hen feather coverage and skin and comb lesions at 59 weeks of age.

Feather Coverage	Diets					SEM	ANOVA *p* Value	Contrasts *p* Values			
	HBP	HBP-I	RBP	RBP-I	RBP-P			Protein	HBP vs. HBP-I	Inositol	RBP-P vs. RBP-I
Neck	2.48	2.44	2.31	2.12	2.33	0.072	0.595	0.151	0.893	0.521	0.380
Wings	3.43	3.43	3.33	3.45	3.47	0.064	0.972	0.761	1.000	0.704	0.921
Back	3.36	3.29	3.14	3.25	3.27	0.087	0.961	0.535	0.818	0.915	0.955
Vent	2.52	2.01	2.55	2.12	2.29	0.097	0.323	0.740	0.108	**0.038**	0.575
Breast	2.70	2.57	2.76	2.54	2.56	0.074	0.875	0.914	0.595	0.334	0.952
Total	14.48	13.74	14.08	13.49	14.97	0.338	0.580	0.624	0.438	0.323	0.143
Lesions											
Skin	0.12	0.13	0.07	0.15	0.12	0.024	0.895	0.873	0.902	0.427	0.666
Comb	2.04	2.00	2.22	1.88	2.10	0.056	0.451	0.808	0.846	0.155	0.241

HBP: High balanced protein; RBP: Reduced balanced protein; I: Inositol; P: Phytase; SEM: Standard error of the mean; Protein: HBP + HBP-I = RBP + RBP-I; Inositol: HBP + RBP = HBP-I + RBP-I. Scoring system per body part: 1 = 0–25% feather coverage; 2 = 25–50% coverage; 3 = 50–75% coverage; 4 = 75–100% coverage.
